# Sources of COVID-19 Vaccine Promotion for Pregnant and Lactating Women in Bangladesh

**DOI:** 10.3390/vaccines11081387

**Published:** 2023-08-20

**Authors:** Berhaun Fesshaye, Sydney A. Wade, Clarice Lee, Prachi Singh, Eleonor Zavala, Hasmot Ali, Hafizur Rahman, Towfida Jahan Siddiqua, Shirina Atker, Ruth A. Karron, Rupali J. Limaye

**Affiliations:** 1 International Vaccine Access Center, Bloomberg School of Public Health, Johns Hopkins University, Baltimore, MD 21231, USA; bfessha1@jh.edu (B.F.); clarice.h.lee@gmail.com (C.L.); prachi@jhu.edu (P.S.); 2 Johns Hopkins School of Medicine, Baltimore, MD 21205, USA; swade9@jhmi.edu; 3 Department of International Health, Bloomberg School of Public Health, Johns Hopkins University, Baltimore, MD 21205, USA; ezavala1@jhu.edu (E.Z.); rkarron@jhu.edu (R.A.K.); 4 JiVitA Project, Johns Hopkins University, Rangpur 8240, Bangladesh; hasmot.jivita@gmail.com (H.A.); hafiz.jivita@gmail.com (H.R.); towfida.jivita@gmail.com (T.J.S.); shirinasumi916@gmail.com (S.A.); 5 Center for Immunization Research, Baltimore, MD 21205, USA; 6 Department of Health, Bloomberg School of Public Health, Behavior & Society, Johns Hopkins University, Baltimore, MD 21205, USA; 7 Department of Epidemiology, Bloomberg School of Public Health, Johns Hopkins University, Baltimore, MD 21205, USA

**Keywords:** vaccines, maternal immunization, COVID-19, trust, Bangladesh, qualitative

## Abstract

COVID-19 vaccines are an effective public health intervention to reduce COVID-19-related morbidity and mortality. Given that pregnant and lactating women have a higher risk of severe COVID-19 complications, it is paramount to understand the factors that inform vaccine decision-making among this population. In this study, we sought to identify facilitators and barriers to COVID-19 vaccine acceptance and vaccine promotion in pregnant and lactating women in Bangladesh. We conducted 40 in-depth interviews with 12 pregnant women, 12 lactating women, and 16 health workers from one urban and four rural communities in Bangladesh. We used a grounded theory approach to identify emerging themes. Our results suggest that health workers and religious leaders played key roles in promoting COVID-19 vaccines in this population. Further, we found that the culture of trust in public health authorities and the existing vaccine infrastructure facilitated vaccine promotion. However, changes in vaccine eligibility and myths and rumors acted as both facilitators and barriers to vaccine promotion within our study. It is crucial that maternal immunization vaccine promotion efforts push pregnant and lactating women toward vaccine acceptance to protect the health of mothers and their babies. Additionally, as new maternal vaccines are developed and licensed, understanding how to best promote vaccines within this group is paramount.

## 1. Introduction

The first COVID-19 case in Bangladesh was reported on 8 March 2020. Since then, there have been more than two million confirmed cases of COVID-19 and almost 30,000 deaths [1]. While there is growing evidence of increased risk of COVID-19 complications among pregnant women in other countries, data on SARS-CoV-2 infections among pregnant and lactating women have not been systematically collected in Bangladesh, with only a few studies estimating the risk of SARS-CoV-2 infection in this sub-group. Two studies conducted in Dhaka noted that pregnant women infected with SARS-CoV-2 were at a higher risk of having preterm births compared to those not infected [2,3]. One study pointed out that SARS-CoV-2 infection during pregnancy may cause moderate to severe disease requiring ICU admission and maternal death in 5% of the cases studied [3]. The results of these studies align with other studies conducted globally [4,5,6].

COVID-19 vaccine administration is an effective intervention for the prevention of severe COVID-19 disease and its associated complications. Vaccine roll-out in Bangladesh began on 27 January 2021, with the mass vaccination program starting on 7 February 2021 [7]. Initially, the mass vaccination program only targeted health workers, frontline workers, and all adults 55 years and older, but eligibility eventually expanded to cover all adults aged 18 and older in October 2021 [7,8]. Notably, pregnant and lactating women of any age were not included in the initial vaccine roll-out due to the lack of data on vaccine safety and efficacy in this population. However, in August 2021, the Bangladesh National Technical Advisory Committee on COVID-19 recommended pregnant and lactating women receive COVID-19 vaccines due to the increasing evidence of the harm of SARS-CoV-2 infection and safety of the COVID-19 vaccine during pregnancy. The Government of Bangladesh subsequently updated their guidelines such that pregnant women could receive COVID-19 vaccines after receiving counseling from a physician and signing a consent form, and lactating women could receive vaccines according to the same guidelines as the general adult population [9].

Maternal immunization, or administering vaccines during pregnancy, is an effective intervention that protects both the mother and the baby from various vaccine-preventable diseases [10]. Despite a plethora of evidence supporting maternal immunization as an evidence-based public health approach, uptake of vaccines during pregnancy around the world is relatively low [10]. The World Health Organization currently recommends tetanus (TT/Td) and influenza vaccines for all pregnant women and COVID-19 vaccines when the benefits outweigh potential risks [10].

Given the important role of COVID-19 vaccines in disease prevention, it is critical to identify factors that influence vaccine decision-making among pregnant and lactating women. Previous research has identified that across various geographical settings, health worker recommendations are one of the most important influences on maternal decision-making broadly and immunization decisions specifically [10,11]. A central reason for historically low uptake of maternal vaccines is safety concerns; studies have found that the use of clear communication with health workers can alleviate these concerns [10,12]. Further, physician recommendation of COVID-19 vaccines was found to double willingness to receive the vaccine among adults in Bangladesh [13]. Religion can also shape vaccine decisions [14]. Bangladesh is a predominantly Muslim country, and while Islam has endorsed the importance of vaccines overall, individual religious and cultural beliefs can affect maternal health, including maternal vaccination [14]. A previous study in Bangladesh revealed that Muslim adults were less willing to receive the COVID-19 vaccine compared to Hindu adults, possibly due to myths about the vaccines not being halal [13].

While there is some data on factors that influence vaccine decision-making among the general population in Bangladesh, there is a paucity of literature on COVID-19 vaccine decision-making among Bangladeshi pregnant and lactating women specifically. In this study, we sought to identify key factors influencing vaccine uptake among pregnant and lactating women in Bangladesh. Additionally, we wanted to understand the key facilitators and barriers to promotion of the COVID-19 vaccine in pregnant and lactating women in Bangladesh. 

## 2. Materials and Methods

In this cross-sectional qualitative study, 12 pregnant women, 12 lactating women, and 16 health workers were interviewed for a total of 40 interviews between April and August 2022. Participants were recruited from five different communities in Rangpur Division in northern Bangladesh: Bamandanga (rural), Damodarpur (rural), Kanchibari (rural), Ramjiban (rural), and Rangpur city (urban).

Semi-structured interview guides were designed by the study team and included questions related to COVID-19 vaccine promotion by health workers as well as facilitators and barriers that influenced the vaccine decision-making process among pregnant and lactating women. Before beginning data collection, pre-testing of interview instruments by members of the target audiences occurred. Semi-structured interview guides are available as Supplementary Material. A two-day training focused on human subject research ethics, and qualitative interviewing was held for data collectors, who all had experience in qualitative research. Participants were recruited either at health facilities or through antenatal care registers available in the five sampled communities in the Rangpur Division. After an initial round of recruitment, snowball sampling was used to recruit additional participants. After confirming interest in participation and reviewing eligibility criteria, informed consent was obtained from all participants. Interviews were conducted in Bangla in semi-private settings in the health facility or at the participant’s home. All interviews were audio-recorded, then later transcribed and translated to English. First, transcripts were first translated into English by a study team member fluent in both English and Bangla. Transcripts were then back-translated by an external translator also fluent in both languages to ensure accuracy and reliability. All data, including audio recordings, were stored on encrypted servers, and only members of the study team had access to the data. 

Data were managed and analyzed by a seven-member team using Atlas.ti. Using a grounded theory approach, the team developed, refined, and finalized a code list over three rounds of open coding. Following agreement on a code list, the team coded the transcripts, holding discussions on emerging themes after coding ~25% and 50% of the transcripts. Two researchers conducted inter-rater reliability with ~10% of the transcripts that neither of them had coded (3 transcripts), with a reliability of 93%. The team then identified themes and sub-themes. This study received ethical approval from Johns Hopkins Bloomberg School of Public Health and Bangladesh Medical Research Council.

## 3. Results

**Figure 1:**
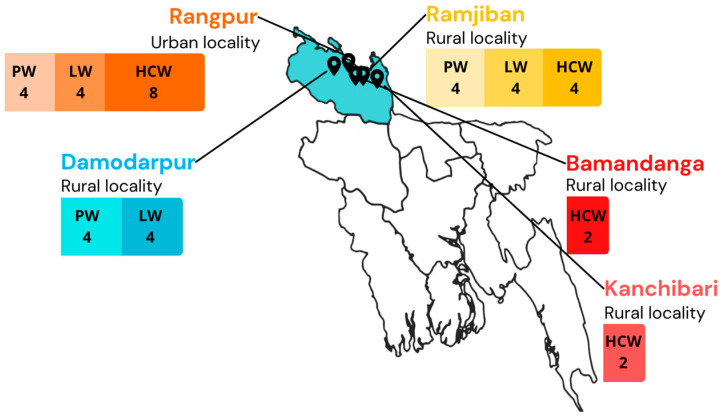
Study population and setting in Bangladesh.

Twelve pregnant women, 12 lactating women, and 16 health workers were interviewed across the five urban and rural localities. See Table 1 for participant information and Figure 1 for study setting.

**Figure 2:**
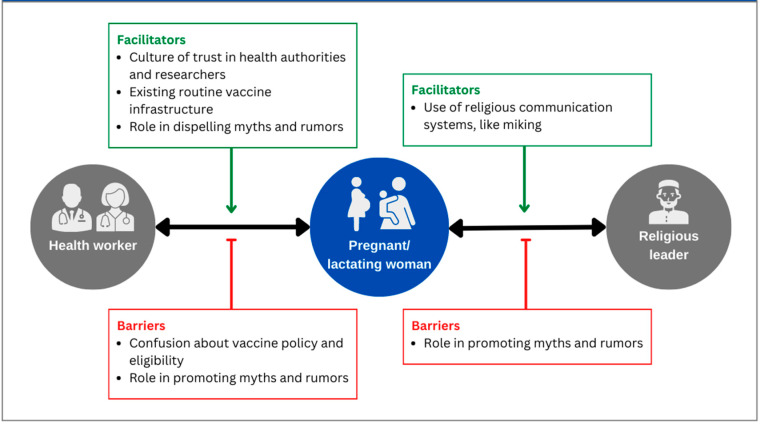
Facilitators and barriers of vaccine promotion to pregnant and lactating women in Bangladesh.

We found that health workers were the primary source of health information among the participants in our study, and that they played an important role in communicating about health information to pregnant and lactating women. We identified two key facilitators that helped health workers promote vaccines among pregnant and lactating women: a culture of trust in public health authorities, and the existence of routine vaccine administration infrastructure. We also found two factors that serve as both facilitators and barriers for health workers to promote vaccines among pregnant and lactating women: vaccine eligibility and clarification related to myths and rumors. Finally, we identified religious infrastructure as playing an important role in vaccine promotion to pregnant and lactating women (Figure 2). In the sections below, we outline each of these themes. 

### 3.1. Role of Health Workers in Communicating Health Information

Health workers were the most prevalent information source for COVID-19 disease, COVID-19 vaccines, routine immunization, and general health for pregnant and lactating women. Health workers were aware of their importance as a source of COVID-19 vaccine promotion for pregnant and lactating women as well as other community members. “More or less all pregnant women know my contact number. They communicate with us over the phone…They ask ‘do you know when I will get Corona vaccine?’” noted a health worker (Health worker, Ramjiban, Rural). One health worker from even discussed the hierarchy of importance among health workers, “But then we saw that the mothers were hesitant, so we requested the medical officers to tell the mothers…But the people who do not know us, or people who know me, but they also know the medical officer, in those cases, the medical officer is given more importance” (Health worker, Bamandanga, Rural).

Given the important role of health workers in promoting the vaccines to pregnant and lactating women, we identified facilitators and barriers related to health worker promotion of the COVID-19 vaccine. Two key facilitators emerged: a culture of trust in public health authorities, and the existence of routine vaccine administration infrastructure. We found two factors that are both facilitators and barriers: vaccine eligibility and clarification related to myths and rumors.

### 3.2. Facilitator: Culture of Trust in Public Health Authorities and Researchers

Health worker willingness to promote the COVID-19 vaccine to pregnant and lactating women was facilitated by a culture of trust in public health authorities and researchers among health workers. Health workers cited public health authorities, including their direct supervisors and other healthcare professionals they reported to, as their primary source of information regarding COVID-19 vaccine policy and guidelines. One health worker stated, “When Corona vaccines first arrived in our Upazila, [the doctors] were our authority. All of them are doctors. All of our authorities are doctors. They were first to give us instructions” (Health worker, Kanchibari, Rural). This reliance on public health authorities for COVID-19 vaccine information facilitated health worker promotion of the COVID-19 vaccine since health workers trusted the positive information from public health authorities about COVID-19 administration in pregnant and lactating women: “After research, it was circulated that the pregnant and breastfeeding women can have the vaccine. Later our supervisors instructed us that now everyone can have the vaccine” (Health worker, Rangpur, Urban). 

Further, several health workers cited trust in COVID-19 vaccine research when asked about why they felt comfortable administering the COVID-19 vaccine to pregnant and lactating women when it became available to them. When asked about the change in pregnant and lactating women’s eligibility for COVID-19 vaccines, one health worker stated, “Their research has shown that pregnant women can have the vaccine and won’t have any trouble. The breastfeeding women can have the vaccine too. Since the research has shown this, so it is established now” (Health worker, Rangpur, Urban). Similar sentiments were expressed by health workers in rural areas as well: “We will not react on [vaccine policy changes] because it’s a matter of research. When they find something in the research, they will instruct as per that” (Health worker, Kanchibari, Rural). 

### 3.3. Facilitator: Existing Routine Vaccine Administration Infrastructure

Prior to the introduction of the COVID-19 vaccine, Bangladesh already had a long-standing community system for routine vaccine administration, such as the tetanus toxoid (TT) vaccine, BCG vaccine, and smallpox vaccine. This existing vaccine infrastructure facilitated promotion of the COVID-19 vaccine to pregnant and lactating women in two ways. First, there was an established trust of health workers for vaccine information among pregnant and lactating women given that health workers routinely provide pregnant and lactating women advice on other vaccines. One health worker stated, “I definitely give advice to pregnant women for TT vaccines, and we see this in every session to every pregnant woman. We educate them about health. We tell them about not only vaccinations but also about antenatal care, family planning, post delivery services, and TT vaccines, everything…the vaccines must be taken” (Health worker, Rangpur, Urban).

Further, health workers often applied the same motivations they had for promoting the TT vaccine to motivate themselves to promote the COVID-19 vaccine. One motivating factor reported by health workers was the satisfaction of knowing their patients did well after receiving the TT vaccine. A health worker shared, “It really gives me pleasure when [pregnant and lactating women] seek vaccination. I really become happy because a mother and a child receive some benefits. Vaccination is an important thing…It reduces the risk of maternal death” (Health worker, Ramjiban, Rural). This sentiment was also expressed by health workers in reference to the COVID-19 vaccine: “The COVID-19 vaccine is sufficient [for pregnant women]…Some mentioned that we have no headache after having Corona vaccine, or we got rid from many old diseases. Now we are healthy, and we are relaxed now. [My patients] discussed this with us. People told me” (Health worker, Ramjiban, Rural).

### 3.4. Facilitator and Barrier: Vaccine Eligibility 

Pregnant women reported health workers as their primary information source to understand their eligibility for services, including immunizations, as one woman stated, “I need to consult with a doctor or need to hear from the clinic that I can take the vaccine during pregnancy…I have to abide by [what doctors say]” (Pregnant woman, Damodarpur, Rural). Another pregnant woman described being explicitly told by a health worker she could not receive a vaccine until she was at least five months pregnant (Pregnant woman, Ramjiban, Rural). One lactating mother discussed how she doubted her eligibility to receive a COVID-19 vaccine before discussing with a health worker: “As my children are feeding milk, I heard that pregnant or lactating women will not be vaccinated. And [the health worker] replied that they are now giving to everyone. Then I went there to get a vaccination” (Lactating mother, Damodarpur, Rural). In some cases, health workers reported being responsible for informing pregnant and lactating women about policy and eligibility changes: “We do miking. They came to know through miking that pregnant and lactating women will get vaccines…We have family planning. Have CSCP. They inform every household member” (Health worker, Kanchibari, Rural).

However, there was confusion about COVID-19 vaccine policy and eligibility for pregnant and lactating women observed among health workers. One health care worker stated that “there is no direction about the pregnant mother … [and] the lactating mother”, clearly indicating a lack of understanding about vaccine guidelines for pregnant and lactating women (Health worker, Bamandanga, Rural). Moreover, many health workers in rural areas relied on instruction and information from higher-level authorities to guide their recommendation of COVID-19 vaccines to pregnant and lactating women. However, it seems there was not clear, direct communication or training from these authorities, which led to a lack of understanding for health workers. When asked if they give vaccines to pregnant mothers or lactating women, a health worker in rural Bamandanga district answered, “They didn’t inform me about that. They are giving vaccines. But the higher authority didn’t inform us why they are giving vaccines or not” (Health worker, Bamandanga, Rural). Another health worker in the same district mentioned, “… we did not get training, or get these instructions in writing (haate kolome paini), they only locally said one day that now we can give it to pregnant women. So if there was more training, then we would understand even better” (Health worker, Bamandanga, Rural). The lack of information trickling down the health care system caused health workers to feel uneasy and uncertain about recommending the vaccine to pregnant and lactating women. 

### 3.5. Facilitator and Barrier: Dispelling Myths and Rumors

Health workers also worked to dispel myths concerning the COVID-19 vaccine, as one pregnant woman reported doctors informing her that vaccines do not cause autism: “When the doctors [informed] I came to know that actually it’s not bad, then I took the vaccine” (Pregnant woman, Damodarpur, Rural).

However, health workers had challenges dispelling myths. A health worker mentioned that “if we give [the COVID-19 vaccine] before 3 months, and they haven’t conceived, then they get their menstruation, they might think that they had an abortion because of the vaccination. And then again if we give the vaccine after 8 months of pregnancy, say the baby is born with some kind of problem, or if the baby is stillborn, they might think that the vaccination is the reason that they lost their baby” (Health worker, Bamandanga, Rural). They also noted that these fears were “true for TT vaccination” as well (Health worker, Bamandanga, Rural). Therefore, some health workers were only administering the COVID-19 vaccine during the second trimester to avoid any misinformation spreading. The health worker stated that “just like TT vaccination, we do the same with corona vaccine” and that “unless it has been 4 months, [they] don’t give the vaccination. And then right before delivery [they] don’t give it. If it has been 7 months, then [they] don’t give the vaccination” (Health worker, Bamandanga, Rural). It was also discussed that health workers “will face opposition and other barriers” if these myths spread, so they only vaccinate if it has been 3 or 4 months (Health worker, Bamandanga, Rural). Furthermore, religious leaders played a role in hindering uptake of COVID-19 vaccine among pregnant and lactating women. According to one lactating mother, “[s]ome say if you take corona vaccines you will have a fever, I mean that time a hujur was teaching us that it’s a scientific method. That hujur told us that he was suffering from fever for three days. After hearing that I didn’t go for [a] second dose” (Lactating mother, Damodarpur, Rural). Given the strong religious infrastructure and influence it has on communities, it is not surprising that religion has both a positive and negative effect on vaccine behaviors.

### 3.6. Role of Religious Infrastructure in Communicating Health Information

Participants also discussed the importance of religion, specifically Islam, in COVID-19 vaccine promotion. One lactating mother discussed that religion has motivated many to receive a vaccine, “There is no one who did not take [the COVID-19 vaccine]. Everyone takes it now…The experience is that, if Allah decides to harm someone, then they will not be able to stop [it]. So the vaccine is out now, you have to take it” (Lactating mother, Rangpur, Urban). Health workers also mentioned using religion as a catalyst for their vaccine promotion efforts. One health worker stated, “And we motivate [community members] by saying ‘we received a vaccine and we are healthy by the grace of Allah, so why don’t you take it?” (Health worker, Ramjiban, Rural). Another health worker described their own vaccine counseling method: “We suggest that pregnant women can also take the vaccine. In the earlier time, they used to get a bit afraid…but we counseled them…that you will also not face any problem by the grace of Allah, so that they take the vaccine” (Health worker, Rangpur, Urban).

A prominent COVID-19 vaccine information source mentioned throughout interviews was miking. Given that Bangladesh is a predominately Muslim country, the use of loudspeakers as a call for prayer at mosques was present long before the pandemic. However, participants discussed that those loudspeakers were adapted by both religious leaders and health workers to promote COVID-19 vaccines. When asked how community members learn about COVID-19 vaccination, in addition to health workers, a pregnant woman from urban Rangpur noted, “They also announce it from the mosque” (Pregnant woman, Rangpur, Urban). A lactating mother discussed what she heard from miking at the mosque, “They said that we have to get vaccines to stay safe otherwise we have to buy the vaccines…That is why for fear of buying it, we took the vaccine” (Lactating mother, Ramjiban, Rural) Different approaches for vaccine promotion were utilized during miking, but overall, participants cited it as a central tool for their vaccine knowledge. 

## 4. Discussion

Although evidence supports maternal immunization as a method to reduce both maternal and infant mortality, uptake of vaccines during pregnancy is sub-optimal [10]. Health worker recommendation has been shown as a central factor for maternal vaccine uptake in various studies [10,11]. Our results further support the placement of health workers as central players in vaccine promotion, given their role as an information source for pregnant and lactating women.

The importance of health workers as an information source on COVID-19 vaccines for pregnant and lactating women was clear from both the health worker’s and pregnant and lactating women’s perspective. Pregnant and lactating women identified health workers as their primary source of health and vaccine information, while health workers also expressed that pregnant and lactating women obtain this information from them. Many pregnant and lactating women were curious about vaccines generally and their eligibility, and after discussing with health workers, many decided to get the vaccine. Moreover, pregnant and lactating women stated that health workers dispelled myths or false information surrounding the COVID-19 vaccine. Since health workers often participated in miking, they were responsible for informing communities, especially pregnant and lactating women, and some health workers stated that pregnant women discussed vaccines with them over the phone.

Given health workers’ significant influence on vaccine behavior, we explored facilitators and barriers of health worker vaccine promotion for pregnant and lactating women. Trust in public health authorities amongst health workers and the existence of routine immunization infrastructure served as facilitators. Health workers cited that they received COVID-19 policy information from public health authorities and trusted the instructions that they were given by these authority figures. Health workers also built on the strong foundation routine immunization programs had in Bangladesh to further promote COVID-19 vaccine uptake.

On the other hand, barriers did exist and posed challenges to encouraging pregnant and lactating women to get vaccinated. Many health workers stated that they were unsure of COVID-19 vaccine policies and lacked clarity from higher-level authorities, possibly delaying vaccine uptake by pregnant and lactating women. Another difficulty health workers faced when promoting the COVID-19 vaccine was vaccine myths and safety concerns. Health workers expressed concern that myths surrounding the tetanus vaccine may transfer or translate to COVID-19 vaccine fears.

Our study found that religion acted as both a facilitator and a barrier in vaccine decision-making. Many pregnant and lactating women had cited that religion had motivated them to get vaccinated, and health workers also encouraged vaccine uptake in the name of Allah. Moreover, many study participants identified miking as a prominent information source for the COVID-19 vaccine. Miking was made possible because of the existing infrastructure–-call for prayer at mosques. Health workers and religious leaders were able to utilize this structure to spread information on the COVID-19 vaccine. However, given religion’s huge impact on the community, fear and myths among religious leaders could dissuade other community members from not getting vaccinated.

We identified three key facilitators and three key barriers to COVID-19 vaccine promotion to pregnant and lactating women in our study. When considering communication of future maternal vaccines, such as an RSV vaccine, it will be important to leverage these facilitators while minimizing these barriers to support vaccine promotion of any new vaccines. Our study demonstrated that health workers rely on guidance from higher-level health authorities and seek out health research that will inform their care for patients. Therefore, a strong top-down communication system that clearly explains the latest vaccine guidelines and research findings is needed to support promotion for new vaccines among local-level health workers. Indeed, prior research has shown that high-quality training for health workers that incorporates curricula on communication skills, vaccine hesitancy, and vaccination competency is critical for health worker promotion of maternal vaccines to pregnant and lactating women [15,16]. Strengthening the top-down communication would also limit the reliance on outdated or inaccurate vaccine policies. In this study, health workers noted that when they were unsure of the vaccine guidelines, they would often apply the guidelines that they use for the TT vaccine. By having a strong top-down communication system that clearly outlines new vaccine guidelines, health workers will not have to rely on inaccurate information when administering any new vaccine.

The influence of religion on vaccine acceptance is another central focus point, especially in Bangladesh, with a predominantly Muslim population that adapted mosque loudspeakers for vaccine promotion throughout the COVID-19 pandemic. In future vaccine campaigns, miking similarly can be harnessed to inform and promote the uptake of maternal vaccines.

Given that many Bangladesh residents trust their religious leaders, there is an opportunity for successful vaccine promotion, but there is also a risk of proliferation of myths and misinformation [17,18]. Islamic leaders have previously worked as positive influences for vaccination behaviors, such as in Saudi Arabia where leaders promoted COVID-19 vaccines, leading to increased uptake [19]. There have also been myths and negative theories spread by Hindu and Muslim religious leaders that have negatively impacted vaccination efforts in some communities [20]. Acknowledging both the positive and negative impact that religious infrastructure and leaders can have on community members should exist as a priority for leaders when considering how to increase and sustain maternal vaccine uptake.

This study is not without limitations. Given this is a qualitative study with a small sample size, the results are not generalizable to a population beyond the scope of this study and may lead to potential bias. Given the unique religious infrastructure in Bangladesh that was harnessed for COVID-19 vaccine promotion, these recommendations may not apply in other settings without similar religious influence. Despite this, this study demonstrates the role of health workers and religious leaders in vaccine promotion to pregnant and lactating women in Bangladesh. Further, we identified four key facilitators and barriers to vaccine promotion that can inform future maternal vaccine initiatives to influence vaccine acceptance. 

## 5. Conclusions

Maternal immunization is a critical intervention for the prevention of morbidity and mortality among mothers and their unborn babies and infants. As shown in our study and in prior research, there are several factors that influence a pregnant or lactating woman’s decision to get vaccinated, including messages from community leaders like health workers and religious leaders. As new maternal vaccines are developed and licensed, such as RSV and GBS vaccines, it is critical that community leaders are leveraged appropriately to promote vaccine acceptance and uptake. Our study demonstrates that adequate training and utilization of trusted communication systems, such as miking for example, would support vaccine promotion to pregnant and lactating women by these leaders. However, additional research is needed to further outline how best to implement these interventions to enhance vaccine promotion and acceptance.

## Figures and Tables

**Table 1:** Sampling by participant type and location.

	Rangpur (Urban)	Damodarpur (Rural)	Ramjiban (Rural)	Bamandanga (Rural)	Kanchibari (Rural)	Total
Pregnant women	4	4	4	0	0	12
Lactating women	4	4	4	0	0	12
Health workers	8	0	4	2	2	16
Total	16	8	12	2	2	40

## Data Availability

The datasets used and/or analyzed during the current study are available from the corresponding author on reasonable request.

## Supplementary Materials

The following supporting information can be downloaded at: https://www.mdpi.com/article/10.3390/vaccines11081387/s1, Supplementary File S1: Semi-Structured Interview Guides. Supplementary material provided is intellectual property of the study team and should not be utilized without permission.
