# Supplementary material for: Sources of COVID-19 Vaccine Promotion for Pregnant and Lactating Women in Bangladesh
Source: Vaccines (Basel). 2023 Aug 20;11(8):1387. doi: 10.3390/vaccines11081387 (PMC10459640; doi:10.3390/vaccines11081387)
Supplement: Supplementary file 1 [file vaccines-11-01387-s001.zip › vaccines-2555232-supplementary.pdf]

JOHNS HOPKINS BLOOMBERG SCHOOL OF PUBLIC HEALTH

DATA COLLECTION FORM FOR KEY INFORMANT INTERVIEWS:

Semi-Structured Interview Guide, Healthcare Providers

Study Title: COVID 19: Preparing Early Adopter Countries for Maternal Vaccination

Principal Investigator: Limaye

IRB No.: IRB00014893

PI Version Date: March 10, 2022 – Version 3

*[**INSTRUCTIONS TO FACILITATOR:** We are very interested in understanding perceptions related to COVID-19 vaccination of pregnant women and women who are currently breastfeeding. The questions below serve as a guide for this discussion. The aim of this interview is to understand the knowledge, attitudes, beliefs, and behaviors surrounding COVID-19 disease and the use of COVID-19 vaccines in pregnant and lactating women in Bangladesh. We aim to speak with a range of stakeholders, including pregnant and lactating/breastfeeding women, their family members, other community members and leaders, healthcare providers, health managers, and policymakers. For this set of interviews, we will focus on healthcare providers, including physicians, nurses, midwives, and others.]*

Opening Script: *Thank you for agreeing to participate in this interview today. Do you agree to be audio recorded?*

GENERAL QUESTIONS

[Notes to facilitator: The first section will help us understand healthcare providers' knowledge and attitudes about COVID-19 disease in their community and how they view the local and national response to control the disease. This will help provide important context to help us better interpret their responses to later questions about vaccination, specifically vaccination of pregnant and breastfeeding women. These questions should be asked of each participant.]

1) I'd like to hear a bit about you.

   Probes:

   - *What is your professional position? Can you tell me a bit about your work experience?*
   - *How long have you been working in the field?*

- *What is your experience in maternal immunization or maternal health? How many pregnant or breastfeeding patients do you regularly see?*

2) Have you ever been ill with COVID-19 or has someone you know ever been ill with this disease?

   Probes:

   - *What have you heard about COVID? [insert local term/language]*
   - *What symptoms did you/they experience?*
   - *How long did you/they feel ill?*
   - *How sick did you/they feel? How severe was your/their illness?*
   - *How did your/their illness impact your/their daily activities and responsibilities?*
   - *[if respondent was never ill with COVID-19]: Do you view COVID-19 as a disease that you may come into contact with during your lifetime or not? Why?*
   - *How does your community or the communities you serve view people who have been ill with COVID?*

3) Do you think that COVID is a problem in your community?

   Probes:

   - *Why or why not?*
   - *Have you seen/treated many COVID-19 patients?*
   - *Do you know of any efforts to control COVID in your community? If so, what are they?*
   - *Do you know what you can do to protect yourself from COVID? Your patients?*
   - *Do you know where you could go if you need help with preventing COVID disease?*

4) Tell me about what your community and/or the government has done to slow the spread of COVID.

   Probes:

   - *Do you think your community/government has done all it can to protect pregnant or breastfeeding women?*
     - *Why or why not?*
   - *Do you think your community/government has done all it can to protect health workers?*
     - *Why or why not?*
   - *Do you think your community/government has done all it can to protect older community members?*

- *Why or why not?*
- *How could the community/government improve their efforts?*

VACCINATION-RELATED QUESTIONS

[Notes to facilitator: This second section focuses on vaccination-related knowledge, attitudes, beliefs, and behaviors. This will help us better understand how healthcare providers access information about vaccines and vaccination, who they trust for that information, what kinds of information or guidance they provide to their patients, and their knowledge and attitudes about the COVID-19 vaccination for themselves and their patients. Most of these questions are about vaccination generally, but some probes will help us better understand knowledge, attitudes, and behaviors specific to vaccination of pregnant or lactating/breastfeeding women. These questions should be asked of each participant.]

This next set of questions is about vaccines. I'd like to know more about your experience with vaccines.

5) Have you ever been vaccinated?

Probes:

- *At what point in your life? Why [if relevant]?*
- *What information sources influence your knowledge and opinions on vaccines?*
- *[If the respondent is pregnant or has had children] Have you ever gotten vaccinated during pregnancy?*
    - *Do you remember which vaccines you received?*
    - *Tell me about your experience getting vaccinated during pregnancy*
- *Some vaccines, like tetanus vaccines, are recommended for pregnant women to help protect both the mother and the baby. What is your opinion on pregnant women getting vaccinated?*

6) I want to ask you a few questions about how you view vaccines as a health worker.

Probes:

- *As you are a health worker, how does your role as one affect your opinion of vaccines, if at all? Meaning, how do you feel about vaccines?*
- *Do you recommend vaccines generally to your patients?*
- *Does your workplace encourage vaccination?*
    - *Among staff? Among patients?*

7) Do you recommend vaccination for your pregnant patients?

Probes:

- *What vaccines do you recommend for pregnant or breastfeeding patients?*
  - *Tetanus?*
- *Where do your pregnant patients gather information on vaccines?*
- *Do your pregnant patients arrive with questions and/or concerns about vaccines?*
  - *How do you typically handle it when a pregnant or breastfeeding patient has concerns about vaccines?*
- *What are the biggest challenges for your patients in accessing vaccines?*
  - *Access to healthcare, distance and time to clinic, cost of transport, supply/availability, cost/willingness to pay*

8) Who do you go to for health information?

Probes:

- *If you were making a decision about vaccines, who would you go to for information?*
- *What about for COVID vaccines? Who would you go to for information?*
  - *Religious leaders?*
  - *Family members? Partner? Mother-in-law?*
  - *Government officials or other community leaders?*
  - *Health workers or community health volunteers?*
- *Do you get information about health and vaccination from media or social media?*
  - *If so, tell me about which sources you rely on.*

9) Have you heard about the COVID vaccine?

Probes:

- *Where did you learn about the COVID vaccine?*
  - *Peers? Professional community? News? Social media? A family member? Somewhere else?*
  - *Do you think this is also where your patients learn about the COVID vaccine?*
- *Do you know where you and/or your patients could get the COVID vaccine?*
- *Do you plan to get the COVID vaccine for yourself? For your family members? Tell me why or why not.*
- *Tell me a bit about whether you will or will not recommend COVID-19 vaccination to your patients? What about to your pregnant or breastfeeding patients? Would you recommend it? Why or why not?*

10) Tell me more about your decision to be vaccinated. If you have not been vaccinated, what are you thinking about in deciding whether or not to get the COVID vaccine? Or, if you have already been vaccinated for COVID-19, what did you think about when you were making that decision?

Probes (if vaccinated):

- *Did you or someone you know have COVID-19? How did that affect your decision to get the COVID vaccine?*
- *What information did you need to make an information decision?*
  - *Who did you talk to?*
  - *What questions did you ask?*

Probes (if not vaccinated):

- *If you had COVID-19 or know someone who did, are you more or less likely to get the COVID-19 vaccine?*
- *If you were able to be vaccinated now, what information do you need to make an informed decision?*
  - *Who would you talk to?*
  - *What questions would you ask?*

Probes (all):

- *Do you know if your family members plan to be vaccinated? Members of your community?*
  - *Tell me a little bit about how the community feels about COVID-19 vaccines.*
- *Do you have any questions about vaccinating pregnant or breastfeeding women with the COVID vaccine?*

COVID-19 VACCINATION QUESTIONS

[Notes to facilitator: This final section focuses on access to COVID-19 vaccines in the community. This includes both the community, generally, and pregnant and lactating/breastfeeding women, specifically. These questions will help us better describe who is being prioritized for COVID-19 vaccination and where community members are accessing the vaccine. They will also help us understand how health workers are or are not being included in COVID-19 vaccination efforts—including vaccination of pregnant or lactating/breastfeeding health workers.]

11) How is the policy for COVID-19 vaccines use in pregnancy and lactation being implemented?

Probes:

- *How did the policy change from not vaccinating pregnant and lactating women to vaccinating pregnant and lactation affect the vaccination campaign? Did this pose any challenges?*
- *How have you responded to this policy change recommending pregnant and lactating women be vaccinated as a health worker?*
- *How did pregnant and lactating women respond to the policy change?*

12) Are pregnant and lactating women (including health care workers) screened out and/or turned away during COVID-19 vaccination distribution campaigns in your community/country? Tell me a little about what you have seen or heard.

    Probes:

    - *How is COVID-19 vaccination information being communicated to pregnant and lactating women? Who is responsible for communicating this information?*

Closing Script: *Thank you very much for participating in this study. Is there anything else you would like to mention before we end the interview? Do you have any questions for me?*

JOHNS HOPKINS BLOOMBERG SCHOOL OF PUBLIC HEALTH

DATA COLLECTION FORM FOR KEY INFORMANT INTERVIEWS:

Semi-Structured Interview Guide, Community Members

Study Title: COVID 19: Preparing Early Adopter Countries for Maternal Vaccination

Principal Investigator: Limaye

IRB No.: IRB00014893

PI Version Date: March 10, 2022 – Version 3

*[**INSTRUCTIONS TO FACILITATOR:** We are very interested in understanding perceptions related to COVID-19 vaccination of pregnant women and women who are currently breastfeeding. The questions below serve as a guide for this discussion. The aim of this interview is to understand the knowledge, attitudes, beliefs, and behaviors surrounding COVID-19 disease and the use of COVID-19 vaccines in pregnant and lactating women in Bangladesh. We aim to speak with a range of stakeholders, including pregnant and lactating/breastfeeding women, their family members, other community members and leaders, healthcare providers, health managers, and policymakers. For this set of interviews, we will focus on pregnant and lactating/breastfeeding women, their family members, religious leaders, community leaders, and other community members.]*

Opening Script: *Thank you for agreeing to participate in this interview today. Do you agree to be audio recorded?*

GENERAL QUESTIONS

[Notes to facilitator: The first section will help us understand participants' family and community lives and their knowledge and attitudes about COVID-19 disease in their community, including how they view the local and national response to control the disease. This will help provide important context to help us better interpret their responses to later questions about vaccination, specifically vaccination of pregnant and lactating/breastfeeding women. These questions should be asked of each participant.]

1) I'd like to hear a little bit about you.

Probes:

- *Where are you from, and where do you live now?*

- *Are you around 18-25? 25-45? 45-60? Older than 60? [capture age range]*
- *Are you married? If so, how long have you been married?*
- *[If pregnant or partner of pregnant woman] Is this your first pregnancy?*
- *[If not first pregnancy] How old are your children?*

2) Tell me about your household.

   Probes:

   - *How many people live in your home?*
   - *How are they related to you?*
   - *Do you have any family members who are pregnant?*
   - *Tell me about what you do during a typical day.*

3) Have you ever been ill with COVID-19 or has someone you know (e.g. close relative, family member, friend) ever been ill with this disease?

   Probes:

   - *What have you heard about COVID? [insert local term/language]*
   - *What symptoms did you/they experience?*
   - *How long did you/they feel ill?*
   - *How sick did you/they feel? How severe was your/their illness?*
   - *How did your/their illness impact your/their daily activities and responsibilities?*
   - *[if never ill]: Do you view COVID-19 as a disease that you may come into contact with during your lifetime or not? Why?*
   - *How does your community view people who have been ill with COVID?*

4) Do you think that COVID is a problem in your community?

   Probes:

   - *Why or why not?*
   - *Do you know of any efforts to control COVID in your community? If so, what are they?*
   - *Do you know what you can do to protect yourself from COVID?*
   - *Do you know where you could go if you need help with preventing COVID disease?*

5) Tell me about what your community and/or the government has done to slow the spread of COVID.

   Probes:

- *Do you think your community/government has done all it can to protect pregnant women?*
    - *Why or why not?*
- *Do you think your community/government has done all it can to protect health workers?*
    - *Why or why not?*
- *Do you think your community/government has done all it can to protect older community members?*
    - *Why or why not?*
- *How could the community/government improve their efforts?*
- *Are there other groups you think the community/government should focus on for protection against COVID-19? If so, can you tell me which groups and why?*

VACCINATION-RELATED QUESTIONS

[Notes to facilitator: This second section focuses on vaccination-related knowledge, attitudes, beliefs, and behaviors. This will help us better understand how community members access information about vaccines and vaccination, who they trust for that information, what barriers to vaccination exist in the community, and their knowledge and attitudes about COVID-19 vaccination. Most of these questions are about vaccination generally, but some probes will help us better understand knowledge, attitudes, and behaviors specific to vaccination of pregnant or lactating/breastfeeding women.]

This next set of questions is about vaccines. I'd like to know more about your experience with vaccines.

6) Have you ever been vaccinated?

Probes:

- *At what point in your life? Why [if relevant]?*
- *[If the respondent is pregnant or has had children]* Have you ever gotten vaccinated *during pregnancy?*
    - *Do you remember which vaccines you received?*
    - *Tell me about your experience getting vaccinated during pregnancy*
- *Some vaccines, like tetanus vaccines, are recommended for pregnant women to help protect both the mother and the baby. What is your opinion on pregnant women getting vaccinated?*
- *If you have heard about vaccination during pregnancy, how did you hear? From whom?*
    - *Do you have any questions about vaccinating pregnant women?*
- *What are the biggest obstacles and challenges learning about and accessing vaccines?*

- *Access to healthcare, distance and time to clinic, cost of transport, supply/availability, cost/willingness to pay*

7) Who do you go to for health information?

Probes:

- *If you were making a decision about vaccines, who would you go to for information?*
- *What about for COVID vaccines? Who would you go to for information?*
    - *Religious leaders?*
    - *Family members? Partner? Mother-in-law?*
    - *Government officials or other community leaders?*
    - *Health workers or [term for CHW]?*
- *Do you get information about health and vaccination from media or social media?*
    - *If so, tell me about which sources you rely on.*

8) Have you heard about the COVID vaccine?

Probes:

- *Where did you learn about the COVID vaccine?*
    - *Did you hear about it from your doctor? The media? A family member? Somewhere else?*
- *Do you know where you could get the COVID vaccine?*
- *Do you plan to get that COVID vaccine for yourself? For your family members? Tell me why or why not.*

9) Tell me more about your decision to be vaccinated. What are you thinking about in deciding whether or not to get the COVID vaccine?

Probes:

- *If you had COVID-19 or know someone who did, are you more or less likely to get the COVID-19 vaccine?*
- *If you were able to be vaccinated now, what information do you need to make an informed decision?*
    - *Who would you talk to?*
    - *What questions would you ask?*
- *Do you know if your family members plan to be vaccinated? Members of your community?*

- *Tell me a little bit about how the community feels about COVID-19 vaccines.*
- *What do you know about vaccinating pregnant women with the COVID vaccine?*
    - *Do you have any questions about vaccinating pregnant women with the COVID vaccine?*

COVID-19 VACCINATION QUESTIONS

[Notes to facilitator: This final section focuses on access to COVID-19 vaccines in the community. This includes both the community, generally, and pregnant and lactating/breastfeeding women, specifically. These questions will help us better describe who is being prioritized for COVID-19 vaccination and where and how community members are accessing the vaccine. They will also help us understand how pregnant or lactating/breastfeeding women are or are not being included in COVID-19 vaccination efforts.]

10) Has your community started to give the COVID-19 vaccine?

Probes (if yes):

- *What groups are currently being vaccinated in your community?*
    - *Do you know when you will be able to get the vaccine?*
- *Do you know anyone who has been vaccinated already?*
    - *Do you know any pregnant women who have been vaccinated?*
    - *[For pregnant women] Has your partner been vaccinated? If not, do they plan to?*
- *[If pregnant] Were you told you could not be vaccinated?*
    - *Do you know why you were told you could not be vaccinated?*
- *[If pregnant partner or family member] Was your pregnant partner/ family member told they could not be vaccinated?*
    - *Do you know why they were told they could not be vaccinated?*

Closing Script: *Thank you very much for participating in this study. Is there anything else you would like to mention before we end the interview? Do you have any questions for me?*
